# EEG Monitoring of Temporal Anticipation in Coincidence Anticipation Timing Tasks: A Scoping Review With Recommendations

**DOI:** 10.1002/brb3.71123

**Published:** 2025-12-10

**Authors:** André Felipe dos Santos, Gabriel Chaves de Melo, Gabriela Castellano, Arturo Forner‐Cordero

**Affiliations:** ^1^ Laboratório De Biomecatrônica, Escola Politécnica Universidade De São Paulo (USP) São Paulo Brazil; ^2^ Instituto De Física Gleb Wataghin Universidade De Campinas (UNICAMP) Campinas Brazil; ^3^ Brazilian Institute of Neuroscience and Neurotechnology (BRAINN) Campinas Brazil

**Keywords:** coincidence anticipation timing, coincident timing task, electroencephalography, motor control, neural mechanisms, temporal anticipation

## Abstract

**Background:**

Coincidence anticipation timing (CAT) tasks require individuals to synchronize their movement with an external moving stimulus. Electroencephalography (EEG), due to its high temporal resolution, offers a valuable tool for investigating the neural processes underlying temporal anticipation in these tasks.

**Objectives:**

This scoping review aims to map the existing literature on EEG monitoring of temporal anticipation during CAT tasks, identify methodological patterns, evaluate the consistency of reported EEG markers, and highlight potential gaps.

**Eligibility criteria:**

Studies were included if they examined EEG activity related to anticipatory processes during CAT tasks in human participants.

**Sources of evidence:**

Studies were obtained from PubMed, Web of Science, and Scopus. A systematic search was conducted in May 2024 and updated in October 2025.

**Charting methods:**

Data were charted across studies, focusing on participant characteristics, protocols, EEG methodologies, and reported outcomes.

**Results:**

Eleven studies met our criterion. Substantial methodological variability was identified in participant setup, task design, EEG acquisition, and data analysis strategies. Although some EEG markers have been recurrently explored, no neural features were consistently assessed across all studies, limiting the identification of robust markers of temporal anticipation. Reporting gaps were observed regarding participant characteristics, anticipation type, and error metrics.

**Conclusions:**

The field remains exploratory, with considerable heterogeneity across studies. To support more reliable comparisons and advance progress, this review proposes practical methodological recommendations focused on standardizing CAT task design and EEG procedures. These guidelines aim to enhance research quality and contribute to a more cohesive understanding of the neural correlates of temporal anticipation.

## Graphical abstract

This scoping review maps EEG studies of temporal anticipation in coincidence anticipation timing (CAT) tasks. Eleven studies were identified, revealing high methodological variability. We provide practical recommendations to standardize CAT task design and EEG procedures, aiming to improve reproducibility and support future research in this emerging field.

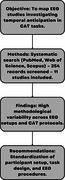

## 1 Introduction

Coincidence‐anticipation tasks, also referred to as coincident timing tasks, require individuals to synchronize their actions with the arrival of a moving stimulus at a specific target point in both time and space (Goodgold‐Edwards 1991). In this review, the term Coincidence Anticipation Timing (CAT) task was used, as we considered it to be the most representative of the topic under discussion. These tasks demand complex cognitive and motor processes, including the detection of movement, judgment, and estimation of the stimulus trajectory, speed, and acceleration to predict arrival time and position, as well as the integration of perceptual information with motor actions to achieve precise timing.

In CAT tasks, participants must anticipate the exact moment when the stimulus will reach its target and prepare to execute a corresponding action at that precise moment, such as pressing a button or intercepting an object (Fleury et al. 1992). Performance is typically evaluated using three error metrics: constant error (CE) for directional accuracy, absolute error (AE) for overall timing accuracy, and variable error (VE) for response consistency (Kim et al. 2013). CAT tasks are used to investigate perceptual‐motor coordination in contexts ranging from everyday activities to sports, rehabilitation, and brain‐computer interface (BCI) development.

Effective performance in such tasks relies heavily on perceptual‐motor skills, particularly the ability to predict and align movements with the anticipated arrival of a stimulus. Poulton (1957) categorized the temporal anticipation process into three distinct types: effector, receptor, and perceptual anticipation. Effector anticipation involves predicting the nature and size of an action required for successful movement execution. Receptor anticipation extends effector anticipation by including the prediction of the duration of the response movement that must be executed. Finally, perceptual anticipation builds upon receptor anticipation by also predicting the future position of the target when the response movement is completed (Poulton 1957).

Understanding the underlying neural mechanisms that facilitate such synchronization is essential given the complexity and precision required in CAT tasks. Electroencephalography (EEG) is particularly well suited for studying these rapid neural processes, as it offers a combination of relatively low cost and portability compared to other brain activity measurement techniques such as functional magnetic resonance imaging (fMRI) and magnetoencephalography (MEG). In terms of spatial resolution, EEG is similar to functional near‐infrared spectroscopy (fNIRS) and inferior to fMRI but offers superior temporal resolution, making it especially suitable for capturing the rapid neural dynamics involved in movement anticipation (Chen et al. 2024). This combination of cost‐effectiveness, portability, and temporal precision positions EEG as an ideal tool for studying the neural processes underlying CAT tasks.

EEG is also the most widely used noninvasive technique for measuring and monitoring the electrical activity of the brain (Müller‐Putz 2020), making it an essential tool for studying and investigating neural activity during various cognitive tasks, including those involving temporal coordination, such as CAT tasks. Its ability to provide valuable insights into how the brain orchestrates the timing of actions before, during, and after movements enables the identification of neural activation patterns related to movement preparation and potential error‐related temporal markers.

Despite the relevance of this research line for understanding motor control, planning, and movement execution, the literature reveals that investigating temporal anticipation in EEG‐monitored CAT tasks is still an emerging field. The limited number of studies and absence of methodological standardization in methods and protocols highlight a knowledge gap. Existing studies employ distinct approaches to data collection, analysis, and interpretation, which limits both comparability and the development of a coherent body of knowledge. This indicates the need for a consensus on the best practices for EEG monitoring in CAT tasks and underscores the importance of more robust and standardized guidelines for future research.

### 1.1 Research Questions and Goals

Given this context, we formulated the following research questions to guide this review. (1) What experimental methods and protocols have been employed to investigate temporal anticipation in EEG‐monitored CAT tasks? (2) Are there consistent EEG signal patterns associated with movement intention and performance in CAT tasks despite methodological variations? (3) What significant gaps exist in the current literature, particularly regarding the standardization of experimental protocols?

To address these questions and map the current state of the field, we conducted a scoping review of studies that have used EEG to investigate temporal anticipation in CAT tasks. This approach was chosen over a systematic review following the guidelines proposed by Munn et al. (2018). Guided by these principles, this review aims to examine how research in this area has been conducted, consolidate existing knowledge, and pursue the following intermediate goals: (1) identify the main experimental methods and protocols employed, (2) synthesize key findings on EEG patterns associated with movement intention in CAT tasks, (3) highlight current gaps in the literature, (4) centralize existing studies to establish a foundation for a potential future systematic review, and (5) propose standardizations and recommendations to guide future research in the field.

## 2 Methods

This scoping review, conducted between 2024 and 2025, was designed in accordance with the Preferred Reporting Items for Systematic reviews and Meta‐Analyses extension for Scoping Reviews (PRISMA‐ScR) guidelines established by Tricco et al. (2018). All 22 applicable items from the checklist were carefully considered to ensure alignment with these standards (Tricco et al. 2018). Although the review followed PRISMA‐ScR recommendations, no formal registration was performed.

### 2.1 Eligibility

The main inclusion criterion for this review, as defined by all the authors, was that studies had to address the analysis of brain processes related to temporal anticipation in humans performing CAT tasks monitored by EEG.

All studies retrieved from the database searches were screened without restrictions on year of publication or language. Consequently, studies published between 1975 and 2025 were covered, and although no language restrictions were applied, all selected articles were ultimately published in English. Furthermore, during the screening process, studies in which task start times were randomized to minimize movement anticipation were excluded, as they did not align with our focus on analyzing anticipatory movements. Only studies that explicitly investigated temporal anticipation were selected for inclusion.

### 2.2 Search Strategy

The electronic databases PubMed, Web of Science, and Scopus were searched for eligible articles related to CAT tasks monitored by EEG. The initial search was conducted on May 9, 2024, and was later updated on October 18, 2025. The search strategy was defined by all authors, with two authors (A.F.S. and G.C.M.) conducting the search. There were no limitations applied in any of the databases. The keywords used were “EEG,” “coincident timing,” “coincidence anticipation,” and their truncated forms (“coinciden* timing” and “coinciden* anticipation”), combined using the Boolean operators “AND” and “OR.” The details of the final strategy for each database are as follows:

- PubMed:
  ○ The search was conducted using PubMed Advanced Search Builder, with the search set to include “All Fields” of the query box.
  ○ Search strategy: (eeg) AND ((coincident timing) OR (coincidence anticipation) OR (coinciden* timing) OR (coinciden* anticipation))
- Web of Science:
  ○ The search was conducted using the Advanced Search tool of the Web of Science website, with the search set to “All” editions of the “Web of Science Core Collection” and including “All Fields” in the fielded search.
  ○ Search strategy: (eeg AND ((coincident AND timing) OR (coincidence AND anticipation) OR (coinciden* AND timing) OR (coinciden* AND anticipation)))
- Scopus:
  ○ The search was conducted using the regular query of the Scopus website, with the search set to include “All Fields.”
  ○ Search strategy: (eeg AND (“coincident timing” OR “coincidence anticipation” OR “coinciden* timing” OR “coinciden* anticipation”))

### 2.3 Review Process and Data Charting

Two authors (A.F.S. and G.C.M.) independently examined all the publications from the three databases and evaluated their titles and abstracts. The articles that were approved at this stage had their full text analyzed for eligibility, also independently by each of these two authors. After this final stage, any divergence between the studies selected by each of the authors was discussed among all the authors and a consensus was reached.

A spreadsheet for data entry on different variables was jointly designed in Excel by two authors (A.F.S. and G.C.M.) after all authors agreed on the variables to be extracted. The first author (A.F.S.) initially mapped the data and discussed the results with the second author (G.C.M.), continuously updating the spreadsheet through an iterative process. Once the data mapping was finalized, the second author (G.C.M.) conducted a detailed review of the spreadsheet to ensure the accuracy and completeness of the collected data. All suggested modifications were discussed and implemented collaboratively, resulting in the final version of the spreadsheet.

All data collected from each article were compiled in this spreadsheet, and any missing variables were flagged to ensure transparency and to facilitate further analysis. The specific data items chosen for this review were intended to provide a complete overview of the methods and results of each study, covering both the quantitative and qualitative aspects. The variables extracted for each included article are listed in Table 1, along with a brief description, when applicable.

**TABLE 1:** Data items extracted from each article.

Authors
Year of publication
Objectives: The main objectives of the study.
Sample description: Characteristics of the sample, including population type, sample size, mean age, and sex.
Participant position requirements: Requirements for the participant's position during the study.
Task description: Basic description of the CAT task performed.
Ocular position: Information on eye position during the task.
Training session: Details on any training sessions provided to participants.
Feedback: Information on feedback given to participants during the experimental session, which refers to all activities excluding the training session.
Type of anticipation: Classification of the type of anticipation involved in the task based on Poulton's framework (Poulton 1957).
Electrode placement: Positions of the EEG electrodes on the scalp.
EEG reference and grounding: Details on the reference and grounding used for EEG recording.
EEG signals analyzed: Specific EEG waves or signal types analyzed in the study.
Electrode impedance: Information on the maximum impedance allowed for the electrodes.
Additional signals measured: Identification of any additional physiological signals measured, if applicable.
Error criteria: Criteria used to define and measure errors in the task.
Main EEG findings: Summary of the key neural activity patterns reported in the study.

### 2.4 Assessment of Methodological Quality

A critical appraisal was conducted to assess the methodological quality of the included studies. To achieve this, a 25‐item checklist was developed based on the 22‐item STROBE (Strengthening the Reporting of Observational Studies in Epidemiology) Statement (Vandenbroucke et al. 2007). As STROBE was originally designed for epidemiological research, its criteria were adapted to suit the context of EEG‐based experimental studies analyzed here.

This adapted checklist consisted of 25 questions, as presented in Table 2 and was used to systematically evaluate the included studies. The evaluation process involved assigning a score to each question, based on the information provided in the articles. A score of 2 was assigned when the study presented sufficient information to answer the question completely. A score of 1 was assigned when the information was incomplete or partially addressed. If the study failed to address the question, it received a score of 0. Questions deemed not applicable to the specific context or design of the article were marked as “NA” and excluded from the denominator of the total score.

**TABLE 2:** Questions based on the STROBE statement.

1. Was the study's design indicated with a commonly used term in the title or abstract?
2. Was the abstract provided with an informative and balanced summary of what was done and found?
3. Were the scientific background and rationale for the investigation explained?
4. Were the specific objectives and hypotheses stated?
5. Were key elements of the study design presented early in the paper?
6. Were the setting, locations, and relevant dates described?
7. Were eligibility criteria and the sources and methods of participant selection provided?
8. Were outcomes, exposures, predictors, confounders, and effect modifiers clearly defined?
9. Were sources of data and methods of assessment described?
10. Were potential sources of bias addressed?
11. Was the study size justified?
12. Were the handling of quantitative variables explained, including possible groupings?
13. Were the statistical methods described in sufficient detail?
14. Were numbers at each stage of the study reported, including reasons for nonparticipation?
15. Were participant characteristics and information on exposures/confounders reported?
16. Were outcome events or summary measures provided?
17. Were unadjusted and, when applicable, confounder‐adjusted estimates provided, along with their precision?
18. Were category boundaries reported when continuous variables were categorized?
19. Were estimates of relative risks translated into absolute risks for a meaningful time period, when relevant?
20. Were other analyses conducted and reported?
21. Were key results summarized with reference to study objectives?
22. Were limitations of the study, including potential biases, discussed?
23. Was a cautious overall interpretation of results provided?
24. Was the generalizability of the results discussed?
25. Were funding sources and the role of funders described?

## 3 Results

### 3.1 Literature Search

**FIGURE 1:**
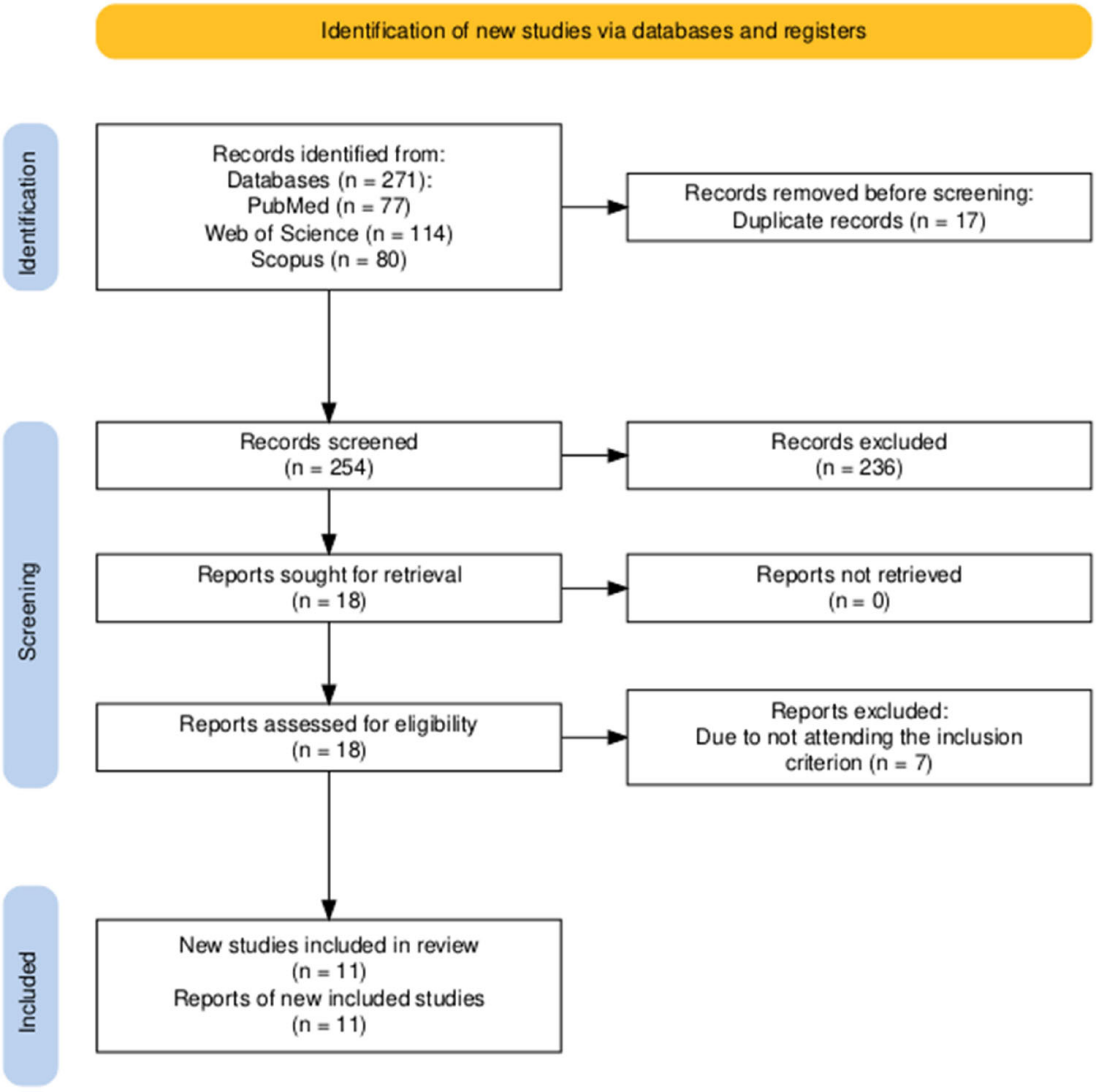
Flow diagram showing the number of studies identified, screened, and included in the review.

The search for articles conducted across the PubMed, Web of Science, and Scopus electronic databases yielded 77, 114, and 80 studies, respectively. After removing the duplicates, 254 studies were identified. These studies were initially screened based on their titles and abstracts, resulting in the exclusion of 236 articles that did not meet the inclusion criterion. The remaining 18 articles underwent full‐text assessment for eligibility, of which seven were excluded for not directly investigating temporal anticipation in CAT tasks monitored by EEG. Finally, 11 studies met the inclusion criterion and were considered relevant for this review (Cui and MacKinnon 2009; Masaki et al. 2012; Nakamoto and Mori 2012; Koshizawa et al. 2013; 2014; de Melo et al. 2020; Liu et al. 2021; Stolz et al. 2023; Tang et al. 2025; Cakir et al. 2025; Zhao et al. 2025). A flow diagram illustrating the selection process is shown in Figure 1 (Haddaway et al. 2022).

### 3.2 Methodological Quality

After the final 11 studies were selected, two authors (A.F.S. and G.C.M.) independently assessed each article and any divergences were discussed between all authors until an agreement was reached. The scoring system developed for this review, inspired by the STROBE statement (Vandenbroucke et al. 2007), allowed for a standardized comparison of methodological quality across studies. This evaluation was based on the 25 questions listed in Table 2, and the results are summarized in Table 3.

**TABLE 3:** Rating score for the assessment of methodological quality from reviewed articles.

Study reference	Question																									Total score	Qualitative percentage
	1	2	3	4	5	6	7	8	9	10	11	12	13	14	15	16	17	18	19	20	21	22	23	24	25		
Cui and MacKinnon 2009	0	2	2	2	2	1	2	NA	2	2	0	2	2	0	NA	2	NA	2	NA	2	2	1	2	0	0	30/42	71.4%
Masaki et al. 2012	0	2	2	2	2	1	2	NA	2	2	0	2	2	2	NA	2	NA	2	NA	2	2	1	2	2	2	36/42	85.7%
Nakamoto and Mori 2012	0	2	2	2	2	1	2	NA	2	2	0	2	2	1	NA	2	NA	2	NA	2	2	1	2	2	2	35/42	83.3%
Koshizawa et al. 2013	0	2	1	2	2	1	1	NA	2	2	0	2	2	1	NA	2	NA	2	NA	2	1	0	2	0	0	27/42	64.3%
Koshizawa et al. 2014	0	2	1	2	2	1	1	NA	2	2	0	2	2	2	NA	2	NA	2	NA	2	2	0	2	0	0	29/42	69.0%
de Melo et al. 2020	0	1	1	2	1	1	1	NA	2	2	0	2	2	2	NA	2	NA	2	NA	2	2	1	0	0	2	28/42	66.7%
Liu et al. 2021	0	2	2	2	2	1	2	NA	2	2	0	2	2	2	NA	2	NA	NA	NA	2	2	0	1	1	2	31/40	77.5%
Stolz et al. 2023	0	2	2	2	2	1	1	NA	2	2	0	2	2	2	NA	2	NA	NA	NA	2	2	2	2	0	2	32/40	80.0%
Tang et al. 2025	2	2	2	2	2	1	2	NA	2	2	0	2	2	1	NA	2	NA	NA	NA	2	2	2	2	2	2	36/40	90.0%
Cakir et al. 2025	2	2	2	2	2	2	2	NA	2	2	2	2	2	2	NA	2	NA	NA	NA	2	2	2	2	2	2	40/40	100.0%
Zhao et al. 2025	0	2	2	2	2	0	2	NA	2	2	2	2	2	2	NA	2	NA	NA	NA	2	2	2	2	2	2	36/40	90.0%

*Note*: The questions were evaluated as 2 = yes, 1 = limited information, 0 = no, and NA = not applicable. Questions evaluated as NA were not considered in the denominator of the total score.

Only two of the 11 studies (Tang et al. 2025; Cakir et al. 2025) explicitly indicated their study design in the title or abstract, obtaining the maximum score for Question 1. Similarly, most studies received a score of 1 for Question 6, indicating a general failure to adequately describe the setting, locations, and relevant dates of their experiments and data collection processes.

On the other hand, Questions 2 (informative and balanced abstract) and 5 (presentation of key design elements early in the paper) were generally well addressed, with only one study (de Melo et al. 2020) scoring 1, suggesting an overall strength in abstract quality and in early methodological clarity.

Although a few studies (Koshizawa et al. 2013; 2014; de Melo et al. 2020) lacked an adequate explanation of their scientific background and rationale (Question 3), all of them clearly stated their specific objectives and hypotheses (Question 4), reflecting consistent competence in their introductions.

For Question 7, concerning eligibility criteria and participant selection methods, most studies provided complete descriptions, while some (Koshizawa et al. 2013; 2014; de Melo et al. 2020; Stolz et al. 2023) failed to describe how participants were sourced or selected, receiving a score of 1.

Questions 8, 15, 17, and 19 were deemed not applicable to the studies included in this review. These items address epidemiological concepts like confounders and relative risks, which are typically not relevant in EEG‐based experimental studies.

Regarding methodological transparency, all studies performed well on Questions 9 (data sources and assessment methods), 10 (potential sources of bias), 12 (handling of quantitative variables), and 13 (description of statistical methods).

A notable gap was observed in Question 11, as only two studies (Tang et al. 2025; Cakir et al. 2025) justified their sample size, resulting in an almost uniform score of 0. Similarly, Question 14 revealed that four studies (Cui and MacKinnon 2009; Nakamoto and Mori 2012; Koshizawa et al. 2013; Stolz et al. 2023) failed to report whether participants were excluded during the study, thereby lacking information on participant flow or exclusion.

It is also worth noting the distinction between Questions 7 and 14; while the former concerns eligibility criteria and recruitment methods, the latter evaluates the reporting of participant numbers at each study stage, including reasons for dropout.

Question 16, regarding the reporting of outcome measures, received a consistent score of 2, indicating that it was well addressed. The same was applied to Question 20, which assessed whether additional analyses were conducted and reported.

For Question 18, five studies (Liu et al. 2021; Stolz et al. 2023; Tang et al. 2025; Cakir et al. 2025; Zhao et al. 2025) received “NA” since they did not categorize continuous variables in their analyses. Question 21, assessing whether results were clearly linked to study objectives, showed strong overall performance, with only one study (Koshizawa et al. 2013) scoring 1.

In terms of study limitations (Question 22), three studies (Koshizawa et al. 2013; 2014; Liu et al. 2021) failed to address them and received a score of 0. Half of the remaining studies (Cui and MacKinnon 2009; Masaki et al. 2012; Nakamoto and Mori 2012; de Melo et al. 2020) received limited scores, while the others (Stolz et al. 2023; Tang et al. 2025; Cakir et al. 2025; Zhao et al. 2025) provided a thorough discussion, receiving a score of 2.

Question 23 asked whether a cautious interpretation of results was provided, and most studies performed well, indicating a measured discussion, with only two studies (de Melo et al. 2020; Liu et al. 2021) receiving 0 or 1. In contrast, Question 24 showed that generalizability was often under‐discussed, with six studies (Cui and MacKinnon 2009; Koshizawa et al. 2013; 2014; de Melo et al. 2020; Liu et al. 2021; Stolz et al. 2023) scoring 0 or 1.

Finally, three studies (Cui and MacKinnon 2009; Koshizawa et al. 2013; 2014) did not report funding sources or clarify the role of funders in the study design (Question 25) and received the lowest score.

Despite these shortcomings, the total methodological scores ranged from 64% to 100%, indicating an overall acceptable quality, particularly given the emerging nature of this research area. It is important to emphasize that no study was, or would be, excluded from this review based on its methodological appraisal. The evaluation was conducted solely for exploratory purposes with the aim of identifying common strengths and recurring weaknesses in methodological reporting across the existing literature.

### 3.3 Data Charted

Following the assessment of the 11 studies, the data charted in the Excel spreadsheet were summarized in Tables 4, 5, and 6. Table 4 provides an overview of each study, including its objectives, sample details, and main findings. Table 5 focuses on the technical specifications of EEG monitoring, facilitating the comparison of methodologies across studies. Table 6 details the specific aspects of CAT tasks, offering insights into the various experimental setups and procedures used. Collectively, these tables offer a comprehensive understanding of the methodologies and findings of the studies.

**TABLE 4:** General characteristics of the included studies.

Study reference	Objectives	Sample description	Main EEG findings
Cui and MacKinnon 2009	Reexamine the comparison of MRPs that precede temporally predictive movements and reactive movements.	A: 9 (7) B: 33 ± 10 C: 9 D: neurologically healthy	RC increased premovement MRP magnitude compared to IC or SP tasks. MRPs were characterized by early slow‐rising negativity at vertex (Cz), positivity at frontal cortex (AFz), and reversal potential at mid‐frontal cortex (Fz). Contralateral sensorimotor cortex showed greater MRP activity.
Masaki et al. 2012	Investigate the brain activities associated with timing control and performance monitoring in a CAT task involving effector and receptor anticipations.	A: 16 (16) B: 20.8 ± 1.2 C: 14 D: normal or corrected‐to‐normal vision and members of a university baseball team	CNV displayed greater frontal activity initially, shifting centrally at later stages. CNV magnitude was enhanced under conditions demanding faster reactions, especially at Cz. N+50 amplitude also increased with higher timing demands. The authors suggest that the CAT task developed, based on gradual force production and considering both receptor and effector anticipations, is useful to explore the kinetic aspects of timing control and the neural processes involved in the preparation and evaluation of timing production.
Nakamoto and Mori 2012	Investigate whether experts in fast‐ball sports exhibit a superior rate of movement reprogramming in CAT tasks with time pressure, and to clarify the specific cognitive activities associated with the higher rate of reprogramming in the experts by means of recording the ERPs.	A: 14 (14) B: not provided (range: 18–23) C: not provided D: college students with normal or corrected‐to‐normal vision, half were experts in fast‐ball sports and the other half (the control group) were members of the track and field team with no specific baseball training	Experts displayed faster N200 latency, enhanced frontal Pd300 amplitude, and delayed P300 latency when target velocity changed unexpectedly, indicating efficient reprogramming of motor skills and enhanced inhibitory control in expert fast‐ball sports athletes compared to track and field athletes.
Koshizawa et al. 2013	Examine beta band patterns in a CAT task with visible and masked sections to determine the role of the cerebral cortex in this task.	A: 10 (10) B: 21.8 ± 0.6 C: 10 D: no history of seizures, psychiatric illness, or severe head injury	Visual masking influenced beta band activity differently across cortical areas: increased beta in Brodmann's areas 6, 7, 19, 39 (during visible section), 19, 37, 40 (first half of the masked section), and decreased in area 17 (first half of the masked section) and areas 9, 10 (second half of the masked section). The authors suggest that Brodmann's areas 7 and 19 indicate involvement in visual attention and target prediction and that masking inactivates Brodmann's areas 9 and 10 possibly affecting response times.
Koshizawa et al. 2014	Examine the effects of training in a CAT task on response time and cortical region activity, in order to determine the precise mechanism of improvement in prediction accuracy.	A: 12 (10) B: 22.5 ± 1.6 C: 12 D: no history of seizures, psychiatric illness, or severe head injury	During the first half of the masked section, training influenced beta band activity, with increased activation in Brodmann's area 6 and decreased activation in area 46. The authors suggest that the inactivation of area 46 may reflect the reduced visual monitoring and the automated information processing. Meanwhile, the activation of area 6 may indicate a shift from processing visual input to predicting target movement. Additionally, the shortened peak latency in area 6 suggests a more efficient processing, which may explain the reduced mean AE after training.
de Melo et al. 2020	Analyze EEG signals during the execution of an upper limb CAT task in order to identify temporal marks related to the start of the movement.	A: 3 (not provided) B: not provided C: 3 D: healthy	Low‐frequency potentials at Fz (0.1–2 Hz) decreased and subsequently increased as alpha band power (8–12 Hz) at C4 approached its minimum. The authors suggest that this temporal coupling reflects a correlation between low delta band potentials and alpha band activity.
Liu et al. 2021	Characterize the cascade of kinematic and electrophysiological activities that contribute to successful target shooting.	A: 20 (not provided after the exclusion of four of the 24 original study participants) B: not provided after the exclusion of four of the 24 original study participants C: 20 D: novice marksmen with normal or corrected‐to‐normal vision, no neurological deficits, no family history of photosensitive epileptic seizures, and no previous experience with the study protocol	Training in a VR marksmanship task led to increased premovement beta band power and more negative contralateral VEPs, indicating improved visual–motor integration and motor learning. After trigger execution, potentials were more negative for errors and resembled an FRN that appeared earlier and with reduced amplitude across practice days. The authors suggest that these findings reflect training‐induced neural adaptations and the utility of MoBI for linking EEG activity with motor behavior in immersive environments.
Stolz et al. 2023	Investigate and understand the fundamental neural processes that control coincidence anticipation.	A: 22 (7) B: 21.14 ± 3.03 C: 22 (self‐reported as being either right‐handed or ambidextrous) D: normal or corrected‐to‐normal vision	VEPs showed distinct occipital, central, and frontal components significantly greater than baseline. Early frontal positive responses (∼150 ms) appeared specifically during outbound trajectories, but not inbound, indicating distinct neural processing for different motion directions. Additionally, a robust late central‐medial positivity (300–500 ms) was modulated by speed, particularly during outbound motion, suggesting increased attentional allocation. The authors suggest that VEPs and behavioral errors can serve as markers of speed and direction tuning in three‐dimensional CAT tasks.
Tang et al. 2025	Explore how temporal context and motor expertise affect TTC estimation accuracy and its neural correlates during speed changes.	A: 56 (36). Case: 28 (18); control: 28 (18) B: Case: 22.21 ± 3.12; control: 20.59 ± 3.17 C: 56 D: normal or corrected‐to‐normal vision and no neurological deficits, history of severe head trauma, substance abuse, or dependence. Half were expert tennis players enrolled in a specialized program with several years of training and competitive experience, while the other half (control group) had no experience with competitive ball games or music and did not engage in regular exercise	Tennis athletes exhibited greater CNV amplitudes than nonathletes, particularly during deceleration in beat contexts, whereas nonathletes showed larger CNV during acceleration. CNV activity was strongest at frontal–midline sites and higher in the right than in the left hemisphere. Across all participants, frontal activation and right‐lateralized CNV activity were observed during all speed changes, regardless of temporal condition.
Cakir et al. 2025	Investigate the effects of physiological and psychological stressors on referees’ time perception and anticipation timing using EEG.	A: 24 (24) B: 24.29 ± 2.89 C: not provided D: healthy football referees, with an average of 4 years of experience and at least 20 officiated matches in the past 2 years	No significant effects of physical effort or crowd noise were observed on theta or alpha power. However, alpha and theta band power differed significantly under Schumann‐frequency binaural stimulation compared to other conditions. No significant differences were found in time perception or anticipation timing, although referees showed slower responses. The authors suggest that the observed behavioral delays may reflect excessive relaxation associated with elevated alpha and theta activity.
Zhao et al. 2025	Explore how tennis experience, interval duration and target speed influence TTC estimation.	A: 55 (37). Case: 28 (17); control: 28 (18) B: Case: 23.11 ± 2.38; control: 22.19 ± 2.54 C: 55 D: normal or corrected‐to‐normal vision and no neurological or physical impairments. Half were expert tennis players with several years of training and competitive experience, while the other half (control group) had no experience with competitive sports or music and did not engage in regular exercise	Tennis athletes showed higher mean CNV amplitudes than novices, and CNV amplitude was greater for suprasecond than subsecond intervals, supporting its role as a neural index of time accumulation. Alpha‐band power was also higher during suprasecond intervals, suggesting that longer durations allow for more efficient cognitive resource allocation and improved timing performance.

*Note*: Under the column “Sample description,” the sample data from each included study were standardized and synthesized using the following identifiers: A: Number of participants in the study (with the number of male participants in parentheses). B: Mean age of the participants, including the standard deviation when available. C: Number of right‐handed participants. D: Other relevant characteristics of the sample, such as information about vision, neurological health, or sports background.

Abbreviations: AE, absolute error; CAT, coincidence anticipation timing; CNV, contingent negative variation; Cz, Fz, etc., EEG scalp electrode positions (according to the 10–20 system); ECG, electroencephalography; ERP, event‐related potential; FRN, feedback‐related negativity; IC, irregularly‐paced cue; MoBI, mobile brain/body imaging; MRPs, movement‐related potentials; RC, regularly‐paced cue; SP, self‐paced; TTC, time‐to‐contact; VEPs, visual evoked potentials.

**TABLE 5:** Technical specifications of EEG monitoring across studies.

Study reference	Electrode placement	Electrode impedance	EEG reference and grounding	Ocular position	EEG signals analyzed	Additional signals measured
Cui and MacKinnon 2009	87 electrodes positioned based on the International 10–20 system with additional interpolated locations.	Below 15kΩ.	Reference: EEG referenced to an electrode placed over the right mastoid process. Grounding: no given information.	Participants were instructed to fix their gaze on a 2 cm^2^ green circle displayed on a computer monitor placed in the center of their field of view and to try to avoid eye blinks and eye movements.	MRPs and SEPs.	EMG and EOG.
Masaki et al. 2012	128 electrodes positioned according to the Biosemi 128‐channel cap.	Not mentioned.	Reference: EEG re‐referenced to average. Grounding: no given information.	Participants were asked to keep their gaze at the initial position of the dividing line (at 12 o'clock) even after the start of the rotation.	ERPs, specifically the CNV and N+50/fpMP waves.	Extensometry, hEOG, and vEOG.
Nakamoto and Mori 2012	nine electrodes positioned according to the International 10–20 system (Fz, F3, F4, Cz, C3, C4, Pz, P3, and P4).	Below 5kΩ.	Reference: EEG referenced to linked earlobes. Grounding: provided by a cap electrode located midway between Fp1 and Fp2.	Participants were encouraged to fix their gaze on a blue LED positioned in the center of the trackway in order to avoid mixing the ocular artifact on the EEG.	ERPs, specifically the N200, P300, Nd200, Pd300, and Nd400 waves.	hEOG and vEOG.
Koshizawa et al. 2013	128 electrodes positioned according to the 128‐channel EGI's Geodesic Sensor Net.	Not mentioned.	Reference: EEG referenced to the vertex electrode of the placement system and re‐referenced to the average reference. Grounding: provided by the Fpz electrode.	Participants were instructed not to move their eyes during the trials.	Beta band patterns (power in 19–23 Hz as a percentage of total power in 3–30 Hz).	EEG only.
Koshizawa et al. 2014	128 electrodes positioned according to the 128‐channel EGI's Geodesic Sensor Net.	Below 70kΩ.	Reference: EEG referenced to the vertex electrode of the placement system for analyzing beta band patterns and re‐referenced to linked‐mastoids (for analyzing ERPs) and to the average reference (for analyzing power spectrum). Grounding: provided by the Fpz electrode.	Participants were instructed not to move their eyes during the trials.	Beta band patterns (power in 13–23 Hz as a percentage of total power in 3–30 Hz) and ERPs.	EEG only.
de Melo et al. 2020	27 electrodes positioned according to the International 10–20 system (Fp1, Fp2, F7, F3, Fz, F4, F8, FC5, FCz, FC6, T7, C3, Cz, C4, T8, CP3, CP1, CPz, CP2, CP4, P7, P3, Pz, P4, P8, O1, and O2).	Not mentioned.	Reference: EEG referenced to the analog average over all signals at each time sample. Grounding: provided by an electrode placed on the right wrist, which remained resting on the knee.	Not mentioned.	CNV, ERD, and ERS.	Motion tracking by video.
Liu et al. 2021	13 electrodes positioned according to the International 10–20 system (F3, Fz, F4, C3, Cz, C4, T5, T6, P3, Pz, P4, O1, and O2).	Below 10kΩ.	Reference: EEG referenced to the mastoids. Grounding: no given information.	Participants had to make eye movements while aiming at a moving target, whose movement occurred randomly between six possible combinations.	Delta, theta, alpha, and beta bands power; VEPs; and FRN.	Motion tracking and hEOG.
Stolz et al. 2023	32 electrodes positioned according to the International 10–20 system.	Below 10kΩ.	Reference: EEG referenced to the FCz electrode. Grounding: no given information.	Participants were encouraged to keep their eyes on the stationary LED target when performing the task.	VEPs.	EEG only.
Tang et al. 2025	10 electrodes positioned according to the International 10–20 system (Fz, F1, F2, F3, F4, FCz, FC1, FC2, FC3, and FC4).	Below 5kΩ.	Reference: EEG referenced to the FCz electrode and re‐referenced to bilateral mastoids (TP9 and TP10). Grounding: no given information.	A brief central fixation cross was presented before each trial, but it is not clear whether participants received additional instructions regarding eye fixation during stimulus presentation.	CNV	hEOG and vEOG.
Cakir et al. 2025	Five electrodes positioned according to the International 10–20 system (AF3, AF4, T7, T8, and Pz).	Not mentioned.	Reference: no given information. Grounding: no given information.	Not mentioned.	Theta and alpha bands power.	Heart rate.
Zhao et al. 2025	64 electrodes positioned according to the International 10–20 system.	Below 5kΩ.	Reference: EEG referenced to the FCz electrode and re‐referenced to bilateral mastoids (TP9 and TP10). Grounding: provided by the AFz electrode.	A brief central fixation cross was presented before each trial, but it is not clear whether participants received additional instructions regarding eye fixation during stimulus presentation.	CNV and alpha band power.	hEOG and vEOG.

Abbreviations: CNV, contingent negative variation; ECG, electroencephalography; EEG, electroencephalography; EMG, electromyography; EOG, electrooculography; ERD, event‐related desynchronization; ERP, event‐related potential; ERS, event‐related synchronization; FRN, feedback‐related negativity; hEOG, horizontal electrooculography; MRPs, movement‐related potentials; SEP, somatosensory evoked potentials; vEOG, vertical electrooculography; VEPs, visual evoked potentials.

**TABLE 6:** Experimental setups and procedures for CAT tasks in the included studies.

Study reference	Participant position requirements	Task description	Training session	Feedback	Type of anticipation	Error criteria
Cui and MacKinnon 2009	Participants seated in a comfortable chair with their right forearm restrained in a brace in a pronated position with the palm of the hand facing downward on the surface of a table.	Ballistic wrist extension movement of the right hand from the neutral position to an angle between 30° and 45°, in three different cueing conditions: SP, in response to an IC, and in response to an RC.	On a different day as the experimental session. Used to train participants to perform the task; elimination of those who were unable to perform the task within the constraints of temporal accuracy; establishment of each participant's 1RTmin.	Movement timing accuracy provided after the completion of 50 trials.	Receptor.	Not specifically defined, however, the individual trials of the RC test were separated into four reaction groups: RC 1, onset early and temporally inaccurate, between −1 s and −1RTmin; RC 2, onset early but temporally accurate, between −1RTmin and 0 s; RC 3, onset late but temporally accurate, between 0 s and +1RTmin; RC 4, onset late and temporally inaccurate, between +1RTmin and 1 s.
Masaki et al. 2012	Participants comfortably rested their right forearm and palm on a flat board, with index finger positioned on a force‐sensitive key, and with a computer monitor placed 100 cm in front of them.	Pressing the force‐sensitive key by flexing the index finger with two possible times until reaching TTP. This should coincide with the instant at which a clocklike rotational stimulus with two possible constant rotation speeds, displayed in the center of the monitor, completed one rotation.	On the same day as the experimental session, at the beginning and in the middle of it, prior to each new TTP condition. Divided into practice tests with feedback, so that participants became confident to correctly produce the required TTP; and pretests without feedback, in which the trained TTP was combined with the rotational stimulus.	Not mentioned.	Effector and receptor.	Three types of timing errors were analyzed: CE, AE, and VE. To calculate these measures, operations were performed involving the rotation time, the TTP, and the participant's stimulus–response interval.
Nakamoto and Mori 2012	Participants comfortably seated in a sound‐attenuated and electrically shielded room at a distance of 100 cm from a horizontal LED trackway, holding a push‐button with their dominant hand.	Pressing the button at the instant a luminous target in apparent motion reached the end of the LED trackway, with the target's speed suddenly being reduced 100, 200, or 300 ms before it reached the end of the track in some of the tests.	On the same day as the experimental session. Used to familiarize participants with the experimental procedures.	Provided the temporal error results for each trial.	Receptor.	Five types of timing errors were analyzed: induced error, CE, AE, VE, and ΔE%.
Koshizawa et al. 2013	Participants seated in a reclining armchair in a quiet, electrically shielded room, holding a push button in their right hand and with a computer display placed 1.3 m in front of them.	Pressing the button with the right thumb at the instant they anticipated the arrival of a visual target moving downward at the end of a stimulus runway with the final two‐thirds masked. After the initial task, a control task was also performed on the same runway, but without masking.	On the same day as the experimental session. Prior to the experimental session, the participants received a set of standardized instructions and performed 10 practice trials of the task.	Not mentioned.	Receptor and perceptual.	Not specifically defined, however, it is mentioned that the average response time was 1573 ± 19 ms during the masked task and 1492 ± 7 ms during the control task. The time for the target to reach the goal was 1500 ms.
Koshizawa et al. 2014	Participants seated in a reclining armchair in a quiet, electrically shielded room, holding a push button in their right hand and with a computer display placed 1.3 m in front of them.	Pressing the button with the right thumb at the instant they anticipated the arrival of a visual target moving downward at the end of a stimulus runway with the final five‐sixths masked.	On different days from the experimental session. Participants underwent a before‐training session and the following day began the training session, in which they practiced the same task for 10 days over 3 weeks receiving the CE as feedback after each block of trials. The experimental session, performing the same task, took place the day after the last training session.	Not mentioned.	Receptor and perceptual.	Two types of timing errors were analyzed: CE and AE.
de Melo et al. 2020	Participants seated in a chair in front of a computer screen, with their arm resting on a table in a predetermined starting position.	Continuous elbow flexion movement to cover a fixed target on the table with the left hand at the instant they anticipated the arrival of a virtual block on the computer screen reaching its final position.	Not mentioned.	Auditory feedback indicating if the trial was successful.	Effector and receptor.	It was considered an error if the participant did not cover the target fixed to the table within ±200 ms of the virtual block reaching its final position.
Liu et al. 2021	Participants stood in the center of a six‐sided CAVE VR system, wearing 3D glasses and holding a firearm‐shaped game controller in their right hand, while their left hand was positioned along the barrel of the controller, stabilizing it.	Pressing a trigger with the right index finger after a digital target was launched in a combination of three possible horizontal directions and two possible heights. Participants had one chance to hit the target with a precision shot, considering speeds, distances, disturbances such as wind, etc. consistent with the laws of physics.	On the same day as the first experimental session. Participants performed the main task on 3 days within 1 week and, on the first day, before starting the main task, they did two practice rounds to familiarize themselves with the task.	After the trigger was pulled, the screen would freeze, displaying the participant's shot location in comparison to the location of the target, their cumulative shot success, and the number of trials remaining.	Effector, receptor. and perceptual.	Two variables were defined to determine performance improvements through practicing the task: shot success and shot error, the former being the percentage of hits on the target; and the latter, the Euclidean distance between the location of the shot and the center of the target.
Stolz et al. 2023	Participants seated in a chair at a distance of approximately 30 cm from the lower end of an inclined light rail, in a darkened room with the light rail as the only source of illumination.	Pressing a button with the right thumb at the instant they anticipated that a luminous target in apparent motion would hit a stationary LED target on the inclined light rail. There were two possible directions and three possible speeds.	On the same day as the experimental session. Prior to the experimental session, the participants practiced several trials to get used to the task, and also performed five practice trials before executing each of the task blocks.	After pressing the button, the last lit LED remained lit for approximately 1 s, providing the participant visual feedback on their accuracy in comparison to the stationary LED target.	Receptor.	Three types of timing errors were analyzed: CE, AE, and VE.
Tang et al. 2025	Participants comfortably seated in front of a computer approximately 60 cm from the screen, with their dominant index finger resting on the “↓” key of the keyboard, and instructed to avoid finger tapping, nodding, or other body movements during stimulus presentation.	Pressing the key at the instant they anticipated that a moving virtual stimulus would bounce and return to its starting location after occlusion, based on its prior visible motion. Stimulus speed could remain constant or change by ±25% before occlusion.	On the same day as the experimental session. Prior to the experimental session, each group performed 12 practice trials with feedback provided after each trial to become familiarized with the task.	Not mentioned.	Receptor and perceptual.	Two types of timing errors were analyzed: absolute bias and delayed response ratio. To calculate these measures, operations were performed involving the estimated and actual TTC values, with the delayed response ratio representing the proportion of trials in which the estimated TTC exceeded the actual TTC.
Cakir et al. 2025	Participants seated on a stationary bicycle ergometer positioned in a quiet, temperature‐controlled laboratory, with data collection performed between 4:00 p.m. and 5:00 p.m.	For the investigation of anticipation timing, participants used a Bassin Anticipation Timer to hit the target while cycling at 6 mph. The study did not describe the action required for participants to register their responses, although, based on the device's typical use, this was likely through a button press.	On the same day as the experimental session. Prior to the experimental session, participants received brief training to familiarize themselves with the procedures and device usage, and then performed three practice trials before the formal anticipation timing measurements.	No feedback was provided during the anticipation timing task.	Probably receptor anticipation, but unclear as the response modality was not specified.	One type of timing error was analyzed: AE.
Zhao et al. 2025	Not mentioned.	Pressing a key at the instant they anticipated that a moving virtual stimulus would bounce and reach the opposite side of its starting location after occlusion, based on its prior visible motion. Stimulus speed could remain constant or change by ±25% before occlusion.	Not mentioned.	Not mentioned.	Receptor and perceptual.	Three types of timing errors were analyzed: CE, AE, and VE. Additionally, a “remembered speed” index was derived as the ratio between estimated and actual TTC.

Abbreviations: 1RTmin, minimum voluntary reaction time; AE, absolute error; CAVE, cave automatic virtual environment; CE, constant error; IC, irregularly‐paced cue; RC, regularly‐paced cue; SP, self‐paced; TTC, time‐to‐contact; TTP, time‐to‐peak force; VE, variable error; ΔE%, delta error.

Tables 4, 5, and 6 present the general characteristics, technical specifications, experimental setups, and procedures of the 11 studies included in this review. The following sections offer a detailed explanation of the findings outlined in each table to enhance clarity and facilitate deeper understanding of the evidence.

#### 3.3.1 General Characteristics

Table 4 provides an overview of the main aspects of each study, including their objectives, demographic, and clinical characteristics of their samples, and main findings. These studies primarily investigated the influence of different experimental conditions on temporal control during motor tasks, particularly focusing on the role of anticipation in these processes. While the specific goals of the studies varied, a common emphasis was placed on understanding the neural mechanisms underlying temporal anticipation and motor control, with a particular interest in how the brain processes the timing required to initiate and execute precise movements in CAT tasks.

The sample descriptions were generally well detailed, with most studies describing their included participants as neurologically healthy with normal or corrected‐to‐normal vision and no history of psychiatric disorders or significant injuries. Right‐handed participants predominated, although some studies also included left‐handed or ambidextrous individuals. Surprisingly, despite the potential relevance of lateralization in motor and cognitive processing, only one study (Cui and MacKinnon 2009) explicitly assessed handedness using a standardized questionnaire, employing a modified version of the Edinburgh Handedness Inventory (Oldfield 1971; Salmaso and Longoni 1985); no other studies reported the use of standardized questionnaires for handedness or any other participant characteristic. Additionally, variations in participants’ sporting experience were noted (Masaki et al. 2012; Nakamoto and Mori 2012; Liu et al. 2021; Tang et al. 2025; Cakir et al. 2025; Zhao et al. 2025), which could potentially influence performance on the tasks.

The EEG findings revealed consistent patterns related to temporal anticipation, motor preparation, visual prediction, and performance monitoring during CAT tasks. Movement‐related potentials (MRPs) and contingent negative variation (CNV) were observed over the frontal and central regions and modulated by task demands and reaction times (Cui and MacKinnon 2009; Masaki et al. 2012; Tang et al. 2025; Zhao et al. 2025). Event‐related potentials (ERPs), including visual evoked potentials (VEPs) and components such as the N200, P300, and feedback‐related negativity (FRN), were sensitive to motion direction, target speed, and performance outcomes, reflecting changes in attentional allocation and motor learning processes (Nakamoto and Mori 2012; Liu et al. 2021; Stolz et al. 2023).

ERPs, including N200, P300, and FRN, were sensitive to stimulus speed changes, visual occlusion, or feedback processing, indicating dynamic adjustments of attentional and performance‐monitoring systems (Nakamoto and Mori 2012; Liu et al. 2021; Stolz et al. 2023).

Frequency‐domain analyses complemented these findings. Studies focusing on beta band activity demonstrated region‐specific modulations linked to visual masking and training, with task‐specific increases and decreases in distinct cortical areas (Koshizawa et al. 2013, 2014). One study reported that low‐frequency potentials at Fz were time‐locked to alpha activity at C4, indicating coordinated oscillatory dynamics during movement initiation (de Melo et al. 2020). Other studies also showed task‐related changes in alpha and theta power under stress or auditory stimulation (Cakir et al. 2025) and increased alpha activity during longer temporal intervals (Zhao et al. 2025), reflecting adjustments in cognitive resource allocation and timing precision.

#### 3.3.2 Technical Specifications of EEG Monitoring

Table 5 explores the technical specifications employed for EEG monitoring across the included studies, comprising details on electrode placement, impedance, referencing and grounding practices, ocular positions, EEG signals analyzed, and any additional signals measured.

Electrode placement varied across studies, with eight studies (Cui and MacKinnon 2009; Nakamoto and Mori 2012; de Melo et al. 2020; Liu et al. 2021; Stolz et al. 2023; Tang et al. 2025; Cakir et al. 2025; Zhao et al. 2025) employing the International 10–20 system, a widely recognized standard in EEG research (Picton et al. 2000). Two studies (Koshizawa et al. 2013, 2014) utilized the same equipment for their different experiments, a 128‐channel system from Electrical Geodesics, Inc. (EGI), and one study (Cui and MacKinnon 2009) used the Biosemi 128‐channel cap. The number of electrodes used varied from low to middle density (5–32 electrodes) in approximately half of the studies (Nakamoto and Mori 2012; de Melo et al. 2020; Liu et al. 2021; Stolz et al. 2023; Tang et al. 2025; Cakir et al. 2025) to high density (87–128 electrodes) in the other half (Cui and MacKinnon 2009; Masaki et al. 2012; Koshizawa et al. 2013; 2014; Zhao et al. 2025).

Low impedance is crucial for ensuring high‐quality EEG signals and minimizing artifacts (Picton et al. 2000). However, only five of the eleven studies (Nakamoto and Mori 2012; Liu et al. 2021; Stolz et al. 2023; Tang et al. 2025; Zhao et al. 2025) explicitly reported maintaining impedances below the commonly recommended threshold of 10 kΩ, with one study (Cui and MacKinnon 2009) using values below 15 kΩ, another below 70 kΩ (Koshizawa et al. 2014), and four studies (Masaki et al. 2012; Koshizawa et al. 2013; de Melo et al. 2020; Cakir et al. 2025) failing to specify the impedance levels used.

Diversity was also observed in the methods used for EEG referencing and grounding. Some studies (Cui and MacKinnon 2009; Nakamoto and Mori 2012; Koshizawa et al. 2013; 2014; Liu et al. 2021; Stolz et al. 2023; Tang et al. 2025; Zhao et al. 2025) employed specific electrode references, whereas others (Masaki et al. 2012; de Melo et al. 2020) used average referencing average referencing. Only one study (Cakir et al. 2025) did not report its referencing method, and grounding information was often unspecified (Cui and MacKinnon 2009; Masaki et al. 2012; Liu et al. 2021; Stolz et al. 2023; Tang et al. 2025; Cakir et al. 2025).

In terms of ocular position, six studies (Cui and MacKinnon 2009; Masaki et al. 2012; Nakamoto and Mori 2012; Koshizawa et al. 2013; 2014; Stolz et al. 2023) instructed participants to minimize eye movements to reduce artifacts in EEG data. One study (Liu et al. 2021) allowed eye movements due to the dynamic nature of the task, whereas two others (de Melo et al. 2020; Cakir et al. 2025) did not address ocular position. Two additional studies (Tang et al. 2025; Zhao et al. 2025) reported the use of a fixation cross before each trial but did not specify whether fixation was required throughout the trials. Typically, participants were instructed to fixate on a specific point to ensure clean and consistent EEG recordings (Cui and MacKinnon 2009; Masaki et al. 2012; Nakamoto and Mori 2012; Stolz et al. 2023).

Despite the limited number of studies in this area, the EEG signals analyzed varied widely across studies, reflecting the broad range of brain activity aspects related to timing and motor control that can be explored in this context. Several studies have also incorporated complementary measurement techniques such as electrooculography (EOG) (Cui and MacKinnon 2009; Masaki et al. 2012; Nakamoto and Mori 2012; Liu et al. 2021; Tang et al. 2025; Zhao et al. 2025), electromyography (EMG) (Cui and MacKinnon 2009), extensometry (Masaki et al. 2012), motion tracking (de Melo et al. 2020; Liu et al. 2021), and heart rate monitoring (Cakir et al. 2025) to enhance the interpretation of EEG data and address potential artifacts.

#### 3.3.3 Experimental Setups and Procedures for CAT Tasks

Finally, Table 6 details the experimental setups and procedures adopted for the execution of the CAT tasks, including participant positioning, task descriptions, presence or absence of training sessions, feedback mechanisms, type of anticipation required, and error criteria applied.

Participant positioning was consistently reported across studies, with detailed instructions provided to ensure controlled experimental conditions. Nine studies (Cui and MacKinnon 2009; Masaki et al. 2012; Nakamoto and Mori 2012; Koshizawa et al. 2013; 2014; de Melo et al. 2020; Stolz et al. 2023; Tang et al. 2025; Cakir et al. 2025) required participants to be seated during the tasks, including one (Cakir et al. 2025) in which participants were seated on a stationary bicycle ergometer while pedaling. One study (Liu et al. 2021) involved a standing position, whereas another (Zhao et al. 2025) did not specify the participant's posture. Several studies (Nakamoto and Mori 2012; Koshizawa et al. 2013; 2014; Liu et al. 2021; Stolz et al. 2023; Cakir et al. 2025) used controlled environments to minimize external interference and ensure high‐quality data collection.

CAT tasks typically required participants to respond accurately when they anticipated the arrival of visual stimuli, with response times and anticipation abilities being the primary metrics of interest. Four studies (Koshizawa et al. 2013; 2014; Tang et al. 2025; Zhao et al. 2025) also included partial masking of stimuli to investigate how anticipation and prediction are affected by limited visual information, adding an additional layer of complexity to the tasks.

Across studies, there was considerable variability in how CAT tasks were implemented; however, some consistency emerged regarding stimulus modality and geometry. One study (Cui and MacKinnon 2009) used an auditory stimulus, whereas 10 (Masaki et al. 2012; Nakamoto and Mori 2012; Koshizawa et al. 2013; 2014; de Melo et al. 2020; Liu et al. 2021; Stolz et al. 2023; Tang et al. 2025; Cakir et al. 2025; Zhao et al. 2025) employed visual stimuli. Among the visual tasks, seven (Masaki et al. 2012; Koshizawa et al. 2013; 2014; de Melo et al. 2020; Liu et al. 2021; Tang et al. 2025; Zhao et al. 2025) presented virtual stimuli (on computer or VR displays) moving horizontally, vertically, or circularly within a plane located in front of the participant. In all these cases, the targets moved across the visual field rather than toward or away from the observer, meaning that time‐to‐contact information (τ) based on changes in visual angle was not present.

The remaining three studies (Nakamoto and Mori 2012; Stolz et al. 2023; Cakir et al. 2025) used physical setups with real light trajectories. One study (Cakir et al. 2025) employed a Bassin Anticipation Timer, although the rail inclination was not reported. Another (Nakamoto and Mori 2012) used a horizontal LED trackway positioned directly in front of the participant, where the target moved from a more distant point toward a nearer one. A third study (Stolz et al. 2023) implemented a vertically inclined light rail positioned in front of the participant, with the target moving from a higher, more distant point toward a lower, nearer one. These last two configurations introduced a small component of motion in the observer's direction, producing gradual changes in the target's apparent size and perceived speed, thereby partially specifying τ. However, none of the studies explicitly reported whether this visual information was considered in their analyses.

Training sessions were conducted in nine studies (Cui and MacKinnon 2009; Masaki et al. 2012; Nakamoto and Mori 2012; Koshizawa et al. 2013; 2014; Liu et al. 2021; Stolz et al. 2023; Tang et al. 2025; Cakir et al. 2025), with training taking place either on the same day as the experiment (Masaki et al. 2012; Nakamoto and Mori 2012; Koshizawa et al. 2013; Liu et al. 2021; Stolz et al. 2023; Tang et al. 2025; Cakir et al. 2025) or on different days (Cui and MacKinnon 2009; Koshizawa et al. 2014). These sessions were generally aimed at familiarizing participants with the task procedures and ensuring their readiness for the experimental phase, with some even including feedback during training to improve participant performance.

Regarding feedback during the experimental sessions, five of the 11 studies (Masaki et al. 2012; Koshizawa et al. 2013; 2014; Tang et al. 2025; Zhao et al. 2025) did not mention providing any feedback; one study (Cakir et al. 2025) explicitly reported that no feedback was provided during the task; and the remaining five studies (Cui and MacKinnon 2009; Nakamoto and Mori 2012; de Melo et al. 2020; Liu et al. 2021; Stolz et al. 2023) employed various feedback strategies depending on the study's objectives. These strategies included visual and auditory cues as well as detailed evaluations during or after the task, reflecting different approaches to enhancing participant performance and task accuracy.

In terms of anticipation type, all studies involved both receptor and effector components, as participants had to perceive target motion and execute a corresponding motor response. However, in most cases (Cui and MacKinnon 2009; Nakamoto and Mori 2012; Koshizawa et al. 2013; 2014; Stolz et al. 2023; Tang et al. 2025; Cakir et al. 2025; Zhao et al. 2025), the action was limited to a small, discrete movement thereby minimizing the role of effector anticipation. As discussed by Masaki et al. (2012), effectively assessing this component requires continuous responses, such as force production, rather than discrete ones like button presses.

Accordingly, four studies (Cui and MacKinnon 2009; Nakamoto and Mori 2012; Stolz et al. 2023; Cakir et al. 2025) were classified as involving only receptor anticipation, whereas two (Masaki et al. 2012; de Melo et al. 2020) integrated continuous force exertion and thus combined effector and receptor anticipation. Four studies (Koshizawa et al. 2013; 2014; Tang et al. 2025; Zhao et al. 2025) used target occlusion paradigms, engaging both receptor and perceptual anticipation as participants inferred the target's trajectory and timing during occlusion. Finally, one study (Liu et al. 2021) incorporated effector, receptor, and perceptual anticipation, offering a complete approach to predicting not only the nature, size, and timing of the response movement, but also the future position of the target during action execution.

Error analysis in CAT tasks is typically conducted using standard metrics, such as CE, AE, and VE. These metrics provide complementary information about the accuracy and consistency of participants’ responses (Kim et al. 2013; Guth 1990).

CE quantifies the systematic bias in timing, indicating whether participants tend to respond earlier or later than the target. It is calculated as the mean signed difference between each response time ($x_{i}$) and the target time (T):

$$CE=\frac{1}{n}\sum_{i=1}^{n}\left(x_{i}-T\right)$$

AE measures the overall accuracy of responses, independent of direction, by averaging the absolute differences between response times and the target:

$$\mathrm{AE}=\frac{1}{n}\sum_{i=1}^{n}\left|x_{i}-T\right|$$

VE indicates the consistency of response times across trials by calculating the standard deviation of response times relative to their own mean ($\bar{x}$):

$$\mathrm{VE}=\sqrt{\frac{1}{n}\sum_{i=1}^{n}{\left(x_{i}-\bar{x}\right)}^{2}}$$

In these equations, $x_{i}$ represents the response time in trial i, T is the ideal target time, $\bar{x}$ is the mean response time, and n is the total number of trials.

Among the 11 included studies, two (Koshizawa et al. 2014; Cakir et al. 2025) reported using only one or two of these standard error measures, whereas four (Masaki et al. 2012; Nakamoto and Mori 2012; Stolz et al. 2023; Zhao et al. 2025) adopted all three in their analyses. The remaining studies (Cui and MacKinnon 2009; Koshizawa et al. 2013; de Melo et al. 2020; Liu et al. 2021; Tang et al. 2025) used alternative or self‐defined criteria for classifying and measuring errors based on response time and performance accuracy.

## 4 Discussion

This scoping review explored the existing literature on EEG monitoring during CAT tasks, focusing on studies that analyzed movement anticipation. Although some of the screened articles involved activities that could be categorized as CAT tasks, such as table tennis, they were excluded if they did not explicitly examine anticipatory processes. By focusing on studies that directly assessed movement anticipation, this review enables a more precise investigation of the neural mechanisms involved in timing and motor control. Moreover, it helps to define the current methodological framework and clarify how anticipation‐related brain activity is measured in the context of EEG and CAT research.

Due to the variability in terminology across publications, such as “Coincident Timing Tasks,” “Coincidence Anticipation Tasks,” and “Coincidence Anticipation Timing Tasks,” and instances where tasks were not explicitly labeled as such, our screening process focused on identifying studies in which the tasks clearly aligned with the CAT framework. This approach allowed us to ensure that only works that specifically targeted movement anticipation were included. The ones that randomized task start times to minimize anticipation or focused exclusively on reactive responses were excluded for not meeting the inclusion criterion. Ultimately, eleven studies that specifically investigated temporal anticipation in CAT tasks through EEG monitoring were selected.

Most studies relied on highly homogeneous participant samples regarding neurological health, visual acuity, and motor ability. Although this choice facilitates experimental control, it significantly limits the external validity of the findings. The near absence of diversity in participant profiles makes it difficult to assess how anticipation‐related neural markers generalize to broader populations, such as older adults, individuals with motor impairments, or those with atypical cognitive profiles. To advance the field, future research must adopt more inclusive sampling strategies.

Another identified problem was the significant methodological diversity in EEG monitoring practices. Differences in electrode placement, number of channels, referencing schemes, grounding procedures, and analyzed signals introduce uncontrolled variability that undermines direct comparisons and reproducibility. Adhering to well‐established EEG practices in electrode montage, impedance control, referencing, and spatial coverage should be prioritized in future research.

Similar inconsistencies were found in the experimental design of CAT tasks. Variations in task descriptions, participant positioning, training sessions, feedback methods, types of anticipation, and error criteria introduce uncontrolled variables that undermine the comparability of the findings. While this diversity reflects the exploratory stage of the field and allows adaptation to specific research goals, it also compromises the development of a coherent understanding of anticipation mechanisms. Establishing a standardized framework for task design and reporting is essential for future progress. Without a shared baseline, it is difficult to build a consistent and cumulative body of knowledge.

### 4.1 Standardizations and Recommendations

The methodological variability identified in this review emphasizes the need for a more consistent and rigorous approach for future EEG‐based CAT research on temporal anticipation. Drawing on the findings presented in Tables 4, 5, and 6, we propose a set of recommendations to improve the comparability, reproducibility, and scientific validity of future studies in this emerging field.

First, greater attention should be paid to the inclusion criteria and characterization of participants, especially concerning individual factors that may influence EEG data and timing‐related performance. In this respect, we recommend incorporating standardized instruments to assess the relevant physiological and behavioral variables. Critically, all studies should assess handedness using a validated tool such as the Edinburgh Handedness Inventory (Oldfield 1971), as well as systematically record participants’ history of neurological or motor disorders, prior experience with similar timing tasks, general visual health, and intake of any psychoactive or neuroactive medications. These factors are central to studies of motor anticipation and EEG signal interpretation, as they can significantly modulate cortical activity. Ideally, such variables should be treated as inclusion or exclusion criteria for participant selection. Otherwise, they must be recorded and incorporated as covariates in statistical analyses.

In addition, we encourage exploratory monitoring of physiological and behavioral conditions known to affect EEG recordings and cognitive performance. For example, the Pittsburgh Sleep Quality Index (Buysse et al. 1989) can be used to evaluate sleep quality, the Morningness–Eveningness Questionnaire (Horne and Ostberg 1976) for circadian preferences, and the International Physical Activity Questionnaire (Craig et al. 2003) for physical activity levels. Logging recent caffeine intake and applying brief objective tasks such as the Psychomotor Vigilance Task (Dinges and Powell 1985) can help to capture fluctuations in alertness. While no reviewed study has yet explored the impact of these variables on EEG signals during CAT tasks, incorporating such measures in future research could help identify systematic sources of interindividual variability, clarify how physiological and behavioral states modulate EEG components, and ultimately refine the identification of neural markers associated with temporal anticipation.

Consistency in participant positioning is also important to ensure high‐quality EEG recordings. Variations in posture, muscle tone, or head orientation can introduce artifacts and compromise the electrode contact stability. We recommend that when applicable, participants be comfortably seated in ergonomically stable positions to minimize muscle activation and ensure consistent signal acquisition. Whenever possible, recordings should be conducted in electrically shielded rooms with minimal auditory and visual distractions. Participants should also be instructed to fixate on a predefined visual target to reduce ocular artifacts. Adhering to these practices lowers inter‐ and intra‐subject variability, improves the quality and reliability of EEG signals, and strengthens the validity of cross‐study comparison. Furthermore, explicitly reporting these procedures enhances the methodological transparency of the study, reinforces the credibility of the dataset, and supports future replications or meta‐analyses.

The role of feedback in CAT experimental designs also requires standardization. Our review found wide variations in whether feedback was provided, the modality employed (e.g., visual displays, auditory tones, or numerical performance indicators), and when it was delivered (e.g., after each trial or at the end of a block). To foster participant engagement, task learning, and performance consistency, we recommend providing immediate trial‐by‐trial feedback using clearly defined modalities aligned with the task objectives. Detailed reporting of feedback strategies is also essential to enable the replication and interpretation of behavioral and neurophysiological outcomes.

The specification of the type of anticipation being investigated is largely underreported across the reviewed studies. Effector, receptor, and perceptual anticipations involve distinct cognitive and motor mechanisms and require specific task configurations, directly influencing both methodological design and theoretical interpretation. We strongly recommend that future studies clearly define and report on specific forms of targeted anticipation.

Significant variability was also observed in electrode placement and channel density. While the flexibility of EEG configurations may be beneficial, the absence of standardized setups hinders data synthesis and prevents reliable cross‐study comparisons. Given the exploratory stage and relative novelty of this field, it remains challenging to establish precise recommendations regarding optimal electrode positioning systems or channel counts. However, to address this gap, and particularly to assist researchers initiating in this area, we recommend adopting a middle‐density electrode montage, specifically, 19 electrodes arranged according to the International 10–20 system. This setup offers a balanced compromise between ease of implementation and signal coverage. By providing a full‐scalp distribution, it enables the capture of a wide range of EEG activity, supporting more diverse analyses without the logistical demands of high‐density arrays. This makes it particularly suitable for early stage research in this field.

Another methodological gap concerns insufficient documentation of referencing and grounding schemes. These technical parameters directly influence EEG signal interpretation and are essential for the reproducibility of analyses. However, several of the included studies, especially regarding grounding, failed to report such details, thus limiting transparency and comparability. Future research should consistently report the reference and ground configurations employed, ideally accompanied by a rationale for their selection.

Although the diversity of EEG signals analyzed reflects the early stage of development of this research field, future investigations may benefit from converging on a set of common EEG markers. This would support meta‐analyses and help to consolidate the understanding of temporal anticipation mechanisms. While current evidence does not allow for precise recommendations regarding the most informative EEG features, this review is expected to serve as a foundation for future studies to identify and refine such markers as the field matures. Meanwhile, we encourage researchers to report their signal choices transparently and consider the exploratory integration of complementary physiological signals, such as EMG, which can support corticomuscular coherence analyses, and EOG, which can verify ocular fixation during tasks. These additions can enrich the datasets, support multimodal analyses, and offer broader insights into sensorimotor coordination.

Finally, standardizing the quantification of errors in CAT tasks is fundamental for aligning future studies with established CAT task literature. The inclusion of the three classical error metrics (CE, AE, and VE) should become routine. These metrics, when clearly defined and consistently applied, support objective and comparable evaluations of the timing accuracy, precision, and performance.

In summary, despite the field being in an early exploratory stage, adopting fundamental methodological standards is both feasible and necessary. The recommendations presented herein aim to support the development of more rigorous, coherent, and comparable studies, contributing to a deeper understanding of the neural correlates of temporal anticipation.

### 4.2 Limitations

This scoping review encountered some limitations. First, although no restrictions were applied regarding publication date, only studies published between 2009 and 2025 met the eligibility criteria. Similarly, although we did not impose language constraints, all included articles were published in English, with only one non‐English study retrieved during the entire screening process. Additionally, despite searching three major scientific databases, the highly specific nature of the topic limited the available evidence, resulting in only 11 studies that met the inclusion criteria.

Furthermore, despite our close adherence to the PRISMA‐ScR guidelines, we did not completely fulfill one non‐optional criterion “Protocol and Registration (PRISMA‐ScR #5),” as we did not register any protocol a priori for this review.

Another limitation was the lack of standardized terminology for CAT tasks across the literature, which made the search more complex and potentially reduced the effectiveness of the study identification process. Given this high terminological variability, we propose adopting CAT as the standard term to refer to this specific class of task to improve clarity, consistency, and future search efficacy in this field.

Despite these limitations, this review systematically mapped the methodological diversity of EEG protocols used to study temporal anticipation in CAT tasks, highlighted the prevalent EEG signal patterns associated with anticipatory motor processes, critically assessed existing gaps in the literature, and proposed standardization guidelines to support the design of future studies in this emerging field.

### 4.3 Conclusions

This scoping review systematically analyzed the current literature on EEG monitoring of temporal anticipation in CAT tasks, guided by three key questions: the diversity of experimental protocols, the consistency of EEG markers across methods, and the main gaps regarding methodological standardization.

Substantial methodological variability was observed across the experimental protocols, complicating direct comparisons between studies. With respect to EEG markers, although certain components and frequency bands were recurrently examined, no single feature was consistently analyzed across all studies. This lack of convergence limits the identification of robust and generalizable neural signatures. Furthermore, several critical gaps were identified, particularly related to participant‐related variables, consistency in participant positioning, feedback protocols, types of anticipation explored, electrode placement and channel density, documentation of referencing and grounding methods, diversity of EEG signals analyzed, and the underuse of standardized error metrics.

To address these limitations, this review proposed a set of practical recommendations aimed at enhancing methodological coherence, improving the quality and comparability of future studies, and supporting the cumulative advancement of knowledge on temporal anticipation mechanisms. Building on these recommendations, the authors plan to design and implement an experimental CAT study based on the proposed methodological framework. This forthcoming work will seek to validate the strategies outlined in this scoping review, and further contribute to the identification of reliable EEG markers associated with temporal anticipation in motor tasks.

## Author Contributions

All authors collaboratively defined the main inclusion criterion and search strategy for the review. A.F.S. and G.C.M. conducted the database search and independently screened all retrieved publications by title and abstract. A.F.S. and G.C.M. also performed full‐text eligibility assessments independently. Discrepancies were resolved through discussion with G.C. and A.F.C. until consensus was reached. All authors agreed on the variables to be extracted. A.F.S. and G.C.M. jointly designed a data extraction spreadsheet in Excel. A.F.S. initially mapped the data and discussed results iteratively with G.C.M., who then performed a detailed review to ensure accuracy and completeness. All suggested revisions were discussed collaboratively, resulting in the final version of the spreadsheet. A.F.S. created Figure 1. After selecting the final eleven studies, A.F.S. and G.C.M. independently analyzed them; any divergences were resolved through group discussion with all authors. A.F.S. wrote the main manuscript text. All authors contributed to interpreting the results, critically revised the manuscript, and approved the final version.

## Funding Information for Included Studies

Various sources of financial support were reported in the studies included in this review. Three studies (Cui and MacKinnon 2009; Koshizawa et al. 2013; 2014) did not mention whether any funding was received. One study (Masaki et al. 2012) reported receiving support from a Grant‐in‐Aid for Scientific Research (C) 21530774 from the JSPS, two Grants‐in‐Aid (the GCOE program and KIBANKEISEI [2010]) from MEXT, and a Waseda University Grant for Special Research Projects (2011A‐091). One study (Nakamoto and Mori 2012) reported receiving a Grant‐in‐Aid for Special Purposes (20509004). One study (de Melo et al. 2020) reported funding from CNPq (Grant 312236/2019‐0), with additional support from CAPES and PIBIC scholarships for two of its authors. Two studies (Liu et al. 2021; Stolz et al. 2023) reported support from the United States Army Research Office (Grant W911NF‐15‐1‐0390). Two studies (Tang et al. 2025; Zhao et al. 2025) reported funding from the Key Laboratory of Exercise and Health Sciences (Shanghai University of Sport), the Ministry of Education Open Fund (2022KF0004), the National Natural Science Foundation of China (No. 32300914), and the Shanghai Pujiang Program (22PJC095). One study (Cakir et al. 2025) explicitly stated that it received no external funding.

## Ethics Statement

The authors have nothing to report.

## Consent

The authors have nothing to report.

## Conflicts of Interest

The authors declare no conflicts of interest.

## Data Availability

The data supporting this review consist of information extracted from previously published articles, which are fully cited and referenced throughout this work.
